# Does supplemental zeolite (clinoptilolite) affect growth performance, meat texture, oxidative stress and production of polyunsaturated fatty acid of Turkey poults?

**DOI:** 10.1186/s12944-018-0820-7

**Published:** 2018-07-28

**Authors:** Emna Hcini, Ahlem Ben Slima, Imen Kallel, Sonia Zormati, Al Ibrahim Traore, Radhouane Gdoura

**Affiliations:** 1 Laboratory of Toxicology and Environmental Health LR11ES06, Sciences Faculty of Sfax, University of Sfax, Sfax, Tunisia; 2 National Laboratory of Public Health, Ouagadougou, Burkina Faso

**Keywords:** Zeolite, Weight of Turkey, Organoleptic parameters, Oxydative stress, Polyunsaturated fatty acid

## Abstract

**Background:**

Following the ban on the use of growth factors, the use of zeolite in poultry feed could be a solution to obtain healthier food products that are more demanded by the consumer.

**Methods:**

Zeolite (Clinoptilolite) was added to turkey male and female feed at concentrations 1% or 2% and was evaluated for its effectiveness on performance of the production. The turkeys were given free and continuous access to a nutritionally non-limiting diet (in meal form) that was either a basal diet or a ‘Zeolite supplemented-diet’ (the basal diet supplemented with clinoptilolite at a level of 1% or 2%).

**Results:**

It was found that adding zeolite in the turkey diet had a positive effect on growth performance and increased weight gain compared to the control. In addition, zeolite treatment had a positive effect on oxidative stress and organoleptic parameters that were measured. It was found that adding zeolite in the turkey diet reduced the MDA level in the liver and in the meat, as compared to the control. Quality of meat was measured as a significantly increase (*p* < 0.05) in pH for male meat, indicated that the zeolite could maintain the quality of longer period. The adding of zeolite in the turkey diet increased level of polyunsaturated fatty acid.

**Conclusion:**

This study showed the significance of using zeolite, as a feed additive for turkey, as part of a comprehensive program to improve growth performance and oxidative stress parameters and to increase level of polyunsaturated fatty acid on the turkey body.

## Background

Nowadays, the consumption of poultry products will be constantly increasing due to the expected evolution of the world population. For this reason, industries seek to improve production to cover this high consumption. Since the interdiction of the using of antibiotic in commercial poultry production, the use of essential oils and fed additives can improve the intake of compound foods [1–6]. Zeolites was used in wide range of agricultural applications and particularly in animal nutrition [7]. Clinoptilolite is a natural zeolite with a particular three-dimensional structure that endows it with specific physicochemical properties that include ion-exchange capacity, absorbency, size-exclusion framework, as well as catalytic properties [8, 9]. Activated and micronized zeolites are detoxifying agents because of their selective binding of heavy metals, O2, and ROS. Various studies have shown that these hydrated aluminosilicates have no toxic effects in either humans or animals [10–12]. Activated and micronized zeolites are used as detoxifying agents in humans. Detoxification is attributed to their ability to reduce lipid peroxidation by scavenging free radicals [13].

Mumpton & Fishman was reported that zeolites have the ability to lose or gain water reversibly and to exchange certain constituent atoms without major change of atomic structure [14]. In this context, Onogi revealed that zeolite should be used to economize on the feed and reduce the moisture content of chicken dropping [15]. Muzaffer et al. suggested that zeolites improves feed efficiency, weight gain of fatting and egg production in laying hens [16]. Moreover, Wu-Haan et al. revealed that zeolites is a beneficial feed additive that exhibits a strong preference for binding nitrogenous cations like NH4 + and NH3 emission [17]. In this context, this substance was recommended to absorb various mycotoxins [13, 18] to reduce ammonia production by pullets [17] and to remove ammonium from aqueous solutions [19], since the high atmospheric ammonia in poultry facilities might damage the respiratory tract lining, or even increased ascites and lower performance [20].

In poultry, transport conditions can influence the quality of the meat stress [21] also; the environmental or behavioral can affect reactive oxygen species (ROS). When the generation of these ROS in a system exceeds the ability of the system to neutralize and eliminate them so oxidative stress develops. Malondialdehyde (MDA) is examples of molecules that can be modified by excessive ROS in vivo and could be used as important and significant biomarkers of oxidative stress [22].

The objective of this study is so to examine the influence of natural zeolites, givens as supplement to feed turkey poults on growth performance, meat texture, oxidative stress and the fatty acid composition.

## Materials and methods

### Turkey management

A total number of 96 forty two- day-old turkey (BUTENA) were selected and divided randomly into three groups housed: control group and two experimental groups (1% zeolite and 2% zeolite), each consisting of 32 turkeys (16 males and 16 females).

Indeed, Feed and water was provided as ad libitum. The experiment was finished when the turkey reached the age of 90 days for females and 110 days for males.

Turkeys received the complete feed mixture BD (0%Z) from the start of the experiment until one week (an adaptation sub-period), followed by the feed mixture BD (0%Z), BD (1%Z) and BD (2%Z) from second week to the end of the experience. All feed mixtures contained the same components; the only difference was that the mixtures designed for the experimental groups (1% zeolite and 2% zeolite) were supplemented with 1 and 2% of natural clinoptilolite (commercial additive ZeoFeed from the company “ROTAMIN”, Turkey).

The basal diet or control diet was a commercial feed that contained yellow corn, soybean meal, calcium carbonate and a mineral-vitamin mixture (Table 1). The turkeys were housed in there compartments with ambient temperature of about 20 °C. Water was provided ad libitum intake throughout the trial period.

**Table 1:** Composition of basal diet (%) and the content of basic nutrients (g/kg)

Nutritional values of diet			
	BD1	BD2	BD3
Composition	%	%	%
Yellow corn	53.00	61.77	69.61
Soybean meal	42.00	34.23	26.39
Vit + Min mixture	5	4	4
Nutrients	%	%	%
Protein	24.01	21.00	18.00
Fat	2.66	2.84	2.98
Energy (Kcal/kg)	2771.85	2870.00	2940.09
Crude cellulose	3	2.84	2.65
Ca	1.25	1.03	1.02
Humidity	12.55	12.83	12.98
Ash	7.11	6.03	5.65

Turkeys received three types of BD (basal diet):

BD1 (basal diet of growth 1) from the start of the experiment until one week (an adaptation sub-period)

BD2 (basal diet of growth 2) from 57 days to 77 days

BD3 (basal diet of finessing) from 78 days to the slaughter

### Data collection

All the turkeys were weighted individually weekly at the same time of day and at the same order of groups to determine the weight gain changes and average daily gain. In addition, feed consumption was measured weekly to estimate the feed conversion ratio.

### Intramuscular ash and fatty acid composition

At the end of the experiment, the turkeys were slaughtered (*n* = 96). Thighs turkey from each groups (10/32, 5 male and 5 female) were selected and harvested at random. The muscular part was used to determine the composition of fatty acids (FA).

In preparation, samples from the 5 male and 5 female from each group were cut from all external fats and were ground by an ultra turrax homogenizer. The total lipids for the analysis of the fatty acids were extracted from alipotent samples of 1 g of the thigh according to Folch et al. [23, 24]. Fatty acid methyl esters (FAME) were prepared, and measured by gas chromatography of FAME in a Chrompack CP 900 apparatus fitted with a flame ionization detector [25]. The results were expressed as a percentage of individual fatty acids in the lipid fraction as described by Pordomingo et al. [20].

### Texture meat color, drip loss and pH 24 h measurement

Texture profile analysis were done on fragments of thigh from each groups (10/32, 5 male and 5 female), stored at least for 24 h at 4 °C. Texture of samples was performed by the determination of Hardness, elasticity and chewiness were using a texturometer (Texture Analyses, TA Plus, LLOYD instruments, England) [26].

Five male and five female samples from each groups (10/32) cleared of fat and cut into a net kept at 4 °C for 24 h were used for determination of the color of meat by a colorimeter.

A section of the pectoralis muscle from five male and five female samples from each groups (10/32) was collected and weighed subsequently. The pectoralis muscle were put in a tightly sealed plastic bag and cooked in water bath at 80 °C for 10 min and the sample were reweighed and drip loss was calculated.

pH value of five male and five female samples from each groups (10/32) was measured after 24 h hours using a solid pH-meter.

### Biochemical assays

#### Measurement of lipid peroxidation levels in meat and liver

Liver and meat homogenate was prepared from each groups (10/32, 5 male and 5 female) to test the enzymatic activity of glutathione peroxidase (GPX), total superoxide dismutase (SOD), catalase (CAT) and content of malondialdehyde (MDA).

#### Malondialdehyde

Lipid peroxidation (LPO) levels in the meat and the liver tissue from five male and five female samples from each groups (10/32) were measured with thiobarbituric acid (TBA) reaction [27]. This method was used to obtain a spectrophotometric measurement of the color produced during the reaction to TBA with malondialdehyde (MDA) at 535 nm.

According to Ben Slima et al. 0.25 ml of sample homogenate was mixed with 0.5 ml of loroacetic acid solution (TCA) and centrifuged at 2500 g for 10 min.0.5 ml of a solution containing 0.67% thiobarbituric acid (TBA) and 0.5 ml of supernatant were incubated for 10 min at 100 °C and cooled. Absorbance of TBA-MDA complex was measured at 532 nm. Lipid peroxidation is expressed as nmoles MDA/g tissue [28].

#### Determination of meat and liver enzymatic antioxidants

The meat and the liver tissue superoxide dismutase (SOD) activities were determined from five male and five female samples from each groups (10/32) by the method of Beauchamp and Fridovich [29]. The reaction mixture contained 50 mM of tissue homogenates in potassium phosphate buffer (pH = 7.4), 0.1 mM methionine, 2 μM riboflavine and 75 μM Nitro Bleu Tetrazoluim (NBT). The developed blue color reaction was measured at 560 nm. Units of SOD activity were expressed as the amount of enzyme required to inhibit the reduction of NBT by 50% and the activity was expressed as U/ mg protein.

Catalase activities were determined in the meat and the liver homogenates, as described by Aebi [30] by the decomposition of hydrogen peroxide and converts it to water and molecular oxygen.

#### Protein determination in meat and liver

According to Lowry et al. the total protein concentration of supernatant was determined using bovine serum albumin BSA as a standard [31].

#### Statistical evaluation

Data analysis was undertaken using SPSS for Windows 20.0 statistical software (SPSS Inc., Chicago, IL, USA).

All results are presented as the mean ± standard deviation (± SD). Means ± S.D. were calculated for normalizing the control as 100%. Differences among treatment and control groups were tested by one way analysis of variance (ANOVA), followed by pair-wise comparisons between group using Student test. *P-*value < 0.05 was considered statistically significant.

Differences at *p* < 0.05 were considered significant.

## Results and discussion

### Measurements of Turkey performance indicators

The effect of zeolite incorporation in the turkey diet on weight gain, on average daily gain and on feed conversion ratio are shown in the Table 2a for female turkey and Table 2b for male turkey. With zeolite, turkey in the experimental group was positively reflected in performance indicators. The results reveal that the average daily gain had increase significantly (*p* = 0.000) both for male and female turkey poults with zeolite. In addition, for male turkey, weight gain in the 2% zeolite group compared to control group was improved significantly (9.36 kg vs. 9.80 kg, *p* = 0.030). Nevertheless, with 1% zeolite there was no significantly different (*p* > 0. 05) for weight gain. Even though, the weight gain tended to be improved for female turkey when zeolite was supplemented to the diets but no significantly different (*p* > 0. 05). In contrary, the differences were not significant (*p* > 0. 05) for the feed conversion ratio for male and female.

**Table 2:** Effect of supplemental zeolite on growth performance of female and male turkey poults

Parameters	BD(0%Z) Mean ± SD	BD(1%Z) Mean ± SD	BD(2%Z) Mean ± SD	*Pa- value*	*Ps- value* 0%Z vs. 1%Z	*Ps- value* 0%Z vs. 2%Z
female						
Initial BW (kg)	1.91 ± 0.05	1.91 ± 0 .04	1.91 ± 0.05	*1.000*	*0.998*	*0.989*
Weight gain ((kg)	5.64 ± 0.41	5.95 ± 0.24	6.08 ± 0.25	*0.120*	*0.194*	*0.079*
Average daily gain (kg)	0.10 ± 0.00	0.10 ± 0.00	0.11 ± 0.00	0.000**	0.007**	0.000**
Daily feed intake (g/bird)	0.34 ± 0.00	0.35 ± 0.00	0.37 ± 0.00	0.000**	0.011	0.000**
Feed conversion ratio	2.31 ± 0.01	2.32 ± 0.01	2.33 ± 0.02	*0.206*	*0.373*	*0.120*
male						
Initial BW (kg)	2.42 ± 0.15	2.38 ± 0.13	2.35 ± 0.08	*0.668*	*0.693*	*0.384*
Weight gain ((kg)	9.36 ± 0.30	9.521 ± 0.20	9.80 ± 0.10	0.027*	*0.373*	0.030*
Average daily gain(kg)	0.12 ± 0.00	0.13 ± 0.00	0.14 ± 0.00	0.000**	0.000**	0.000**
Daily feed intake (g/bird)	0.43 ± 0.00	0.46 ± 0.00	0.48 ± 0.00	0.000**	0.000**	0.000**
Feed conversion ratio	2.33 ± 0.01	2.36 ± 0.01	2.36 ± 0.03	*0.152*	*0.084*	*0.104*

BD basal diet, *Pa- value P-*value ANOVA, *Ps- value P*-value t-test student

*: to mention a significance difference between the mean/SD values and **: highly significant difference

Feed intake was significantly increase by zeolite withe 1% or 2%, even for male and female, so the diet consumed by these two groups was significantly more than that of the control groups (Table 2a, b).

In this study and according to Shariatmadari et al., the zeolite has a positive effect on growth performance of broiler [32]. These results are consistent with those of Eleroglu et al. and Karamanlis et al. in which it indicate that zeolite added to broiler feed increased body weight and growth rate performance [33, 34]. According to Mallek et al., the average growth of broilers were significantly (*p* < 0.05) different between different groups; broilers that were fed on the ‘zeolite diet’ were growing to a faster rate (*p* < 0.05) compared with those of the control group [35]. However, suchy et al. indicate that zeolite increased the feed conversion ratio [36] which not agree with the studies of Jand et al. and Ortatatli et al. [37, 38]. This positive effect of zeolite reported by suchy et al. can be explained by the ability of zeolite to reduce the toxic effects of materials such as aflatoxins [5, 6, 36].

According to Mallek et al., the average growth of broilers were significantly (*p* < 0.05) different between different groups; broilers that were fed on the ‘zeolite diet’ were growing to a faster rate (*p* < 0.05) compared with those of the control group [35]. In addition, with 1% zeolite broilers were significantly heavier from those fed the basal diet at the age of 45 days [37].

### Intramuscular fatty acid composition

In the present experiment, addition of the zeolite has not significantly (*p* > 0. 05) affect on the centration of saturated and poly-unsaturated fatty acids in intramuscular fat (Table 3). However, an increase of the oleic acid (C 18:1) was observed (*p* = 0.005) between control group and 1% Zeolite group (Table 4). However, a decreased of the percentage of palmitoleic acid C: 16:1 (p > 0. 05) was observed without reaching the significance rate. These results may be due of a reduction in Δ9 desaturase activity. This hypothesis could be confirmed by the demonstration of the decrease of mRNA expression level of the Δ9 desaturase gene using quantitative-qPCR. Juárez et al. have reported similar results. There is also a significant decrease in the percentage of the Lignoceric acid (C24:0) (*p* = 0.034) [39]. Mallek et al. was reported similar result about the effects of zeolite on two monounsaturated fatty acids (MUFA) [35]. Zeolite was increased the percentage of 18:1 oleic acid and however decreased 16:1 palmitoleic acid percentage, likely because of a reduction in Δ9 desaturase activity.

**Table 3:** Effect of supplemental zeolite on meat Fatty acids (%) of turkey poult

Fatty acids (%)	BD(0%Z)	BD(1% Z)	BD(2% Z)	*P value*
Saturated fatty acids (SFA)				
Palmitic acid (C16:0)	31.76 ± 4.43	30.85 ± 1.87	29.52 ± 1,03	*0.645*
Stearic acid (C18:0)	12.84 ± 2.86	10.77 ± 2.83	11.58 ± 1.10	*0.599*
Lignoceric acid (C24:0)	3.00 ± 0.78	1.63 ± 1.56	1.41 ± 0.38	*0.203*
Mono-unsaturated fatty acids (MUFA)				
Palmitoleic acid (C16:1)	5.83 ± 0.84	6.54 ± 1.23	4.91 ± 1.81	*0.394*
Oleic acid (C18:1)	24.97 ± 1.53	30.22 ± 0.40	27.16 ± 3.03	0.047*
Poly-unsaturated fatty acids (PUFA)				
Linoleic acid (C18:2)	21.56 ± 1.70	19.32 ± 2.17	24.17 ± 3.03	*0.085*

**Table 4:** Effect of supplemental zeolite on meat Fatty acids (%) of turkey poult (test student)

Groups	*Ps- value* 0%Z vs. 1% Z	*Ps- value* 0%Z vs. 2%Z	*Ps- value* 1%Z vs. 2%Z
Fatty acids			
C16:0	*0.758*	*0.442*	*0.345*
C16:1	*0.460*	*0.467*	*0.267*
C18:0	*0.424*	*0.518*	*0.668*
C18:1	***0.005***	*0.327*	*0.221*
C18:2	*0.233*	*0.210*	*0.064*
C24:0	*0.244*	0.034*	*0.832*

*BD* basal diet, *Ps- value P*-value t-test student

*: a significance difference between the mean/SD values

In addition zeolite reduced the level of total saturated fatty acids (SFA) in intramuscular fat but without reaching the significance rate (*p* > 0.05). This decrease was attributed to the reduction in 16:0 palmitic acid and in 18:0 stearic acid levels. The increase of the level of poly-unsaturated fatty acid could be the direct result of the decrease level of SFA.

It is important to note that PUFAs (Linoleic Acid) have displayed protection against lipid peroxidation increasing the levels of several cellular antioxidants [40].

### Effect of zeolite addition on textural parameters of thigh muscle

**Fig. 1:**
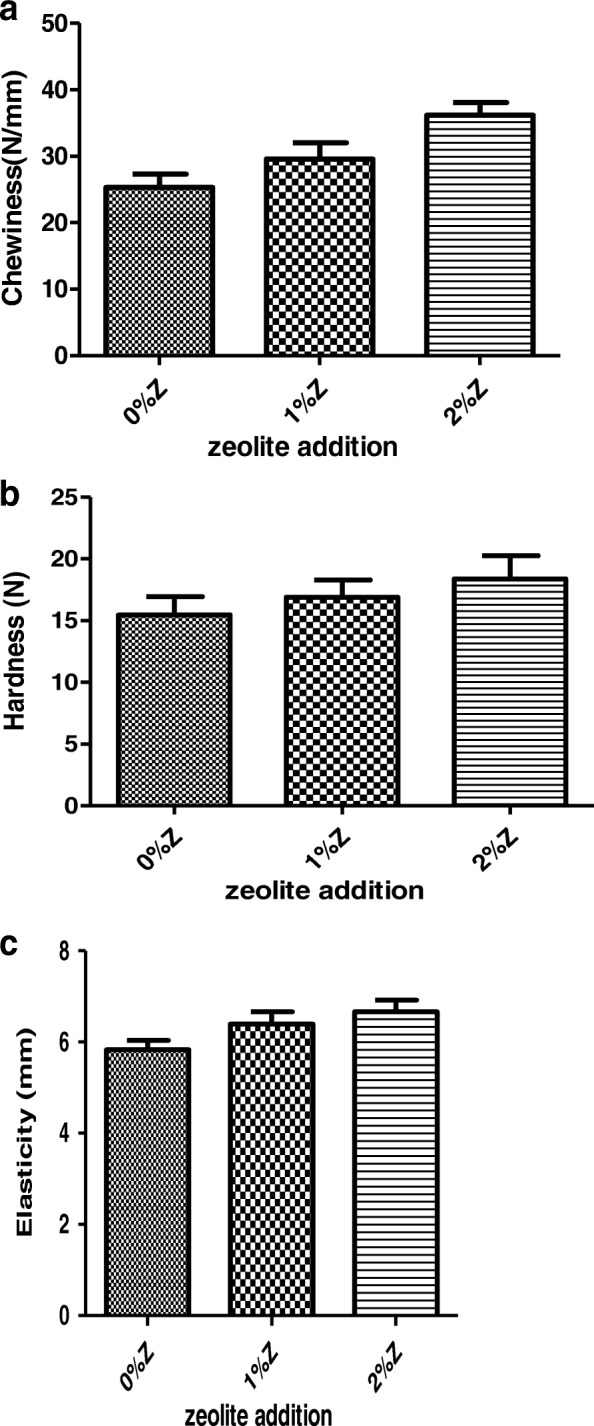
Effect of zeolite addition on textural parameters of thigh muscle female. **a**: chewiness, **b**: hardness, **c**:elasticity

**Fig. 2:**
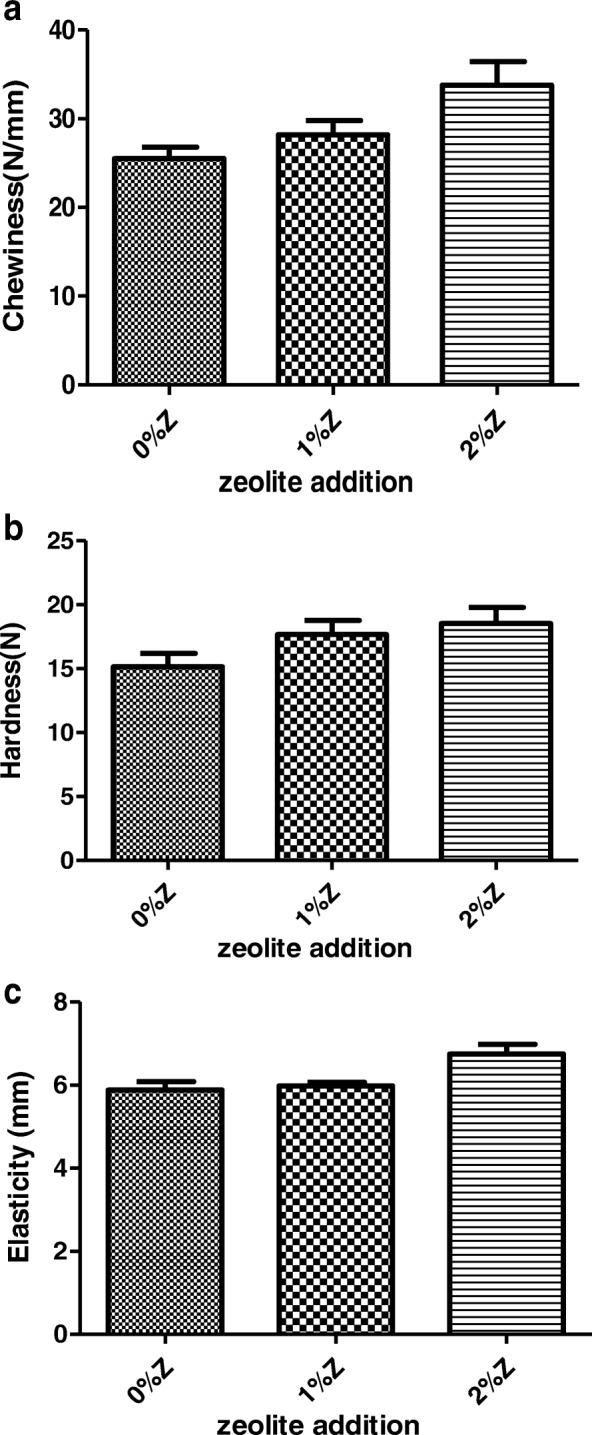
Effect of zeolite addition on textural parameters of thigh muscle male. **a**: chewiness, **b**: hardness, **c**:elasticity

The evolution of textural parameters of the thigh muscle as a function of the level of zeolite added is shown in fig. 1 for female meat and fig. 2 for male meat. The addition of 2% of zeolite was shown that chewiness increases significantly (*p* = 0.004 for female and *p* = 0.024 for male) compared to control group. In addition, elasticity was also increases significantly with 2% of zeolite, from 5.83 to 6.99 for female meat (*p* = 0.034) and from 5.88 to 6.75 for male meat (*p* = 0.022). In contrast, hardness was also increased but without reach significance. Such an evolution of textural parameters can be explained by the change in the water holding capacity. When zeolite was added, a reduction in the compactness of protein gel network allows more binding of water and makes meat tender.

The influence of the presence of natural zeolite on the gelling process of the protein may be the reason of these changes in texture. According to Mallek et al. (2012), zeolite-muscle proteins interaction leads to a change in texture and microstructure of the formulated thigh [35].

### Effect of zeolite addition on meat color, drip loss and pH 24 h

The effect of zeolite on drip loss, pH 24 h, and CIELab color of turkey breast meat booth for female and male is shown in Table 5 (a, b). The supplement of 2% of zeolite significantly increased the pH 24 h (*p* < 0.05) compared to control group only for the breast meat of male (5.80 ± 0.08 vs. 5.94 ± 0.08). However, there were no significant differences (*p* > 0.05) for drip loss, lightness L*, redness a* value and yellowness b* value among the three groups.

**Table 5:** Effect of zeolite addition on meat color, drip loss and pH 24 h for female (3a) and male (3b) meat

(3a): Parameters	BD(0%Z) Mean ± DS	BD(1%Z) Mean ± DS	BD(2%Z) Mean ± DS	*Pa- value*	*Ps-value* 0%Z vs. 1%Z	*Ps- value* 0%Z vs. 2%Z
DRIP LOSS	18.10 ± 1.14	18.08 ± 0. 24	18.10 ± 1.04	*0.999*	*0.978*	*0.997*
pH 24 h	5.78 ± 0.08	5.81 ± 0.09	5.86 ± 0.12	*0.476*	*0 .257*	*0 .579*
L*	56.47 ± 0.97	56.16 ± 1.22	56.59 ± 1.42	*0.848*	*0.670*	*0.876*
a*	7.43 ± 0.53	7.94 ± 0.77	7.94 ± 1.15	*0.574*	*0.261*	*0.398*
b*	11.96 ± 0.91	11.78 ± 0.98	12.23 ± 0.98	*0.756*	*0.772*	*0.658*
(3b): Parameters	BD(0%Z) Mean ± SD	BD + 1%Z Mean ± SD	BD + 2%Z Mean ± SD	*Pa- value*	*Ps-value* 0%Z vs. 1%Z	*Ps- value* 0%Z vs. 2%Z
DRIP LOSS	22.77 ± 1.14	22.97 ± 0.80	22.95 ± 0.55	*0.921*	*0.754*	*0.755*
pH 24 h	5.80 ± 0.08	5.83 ± 0.09	5.94 ± 0.08	*0.062*	*0.613*	***0.028#***
L*	58.19 ± 0.62	57.62 ± 0.77	57.35 ± 0.96	*0.275*	*0.236*	*0.140*
a*	7.94 ± 0.48	7.98 ± 0 .73	8.10 ± 0.58	*0.913*	*0.914*	*0.649*
b*	11.71 ± 0.60	11.46 ± 0.74	11.75 ± 0.57	*0.743*	*0.571*	*0.918*

*BD* basal diet, *Pa- value P-*value ANOVA, *Ps-value P-*value t-test student

*#*: *a* significance difference between the mean/SD values

An increase in pH indicated that the zeolite could maintain the quality of longer period. In the literature, the variation of food additives, animal management and slaughter environment could result in different pH values of the meat. Dietary supplementation with citrus pulp (10%) appeared to decrease the ultimate pH of broiler meat [3]. On the other hand, Jang et al. [4] have reported that supplementation with a mixture of dietary herb extracts (mulberry leaf, Japanese honeysuckle and goldthread) increased the pH values of broiler meat directly after slaughter [4]. However, feeding a diet supplemented with oregano (3%) (Young et al.), grape seed extract (2500 ppm), green tea extract (2500 ppm) (Rababah et al.), or a cranberry extract at concentrations between 40 and 160 mg kg^−^ 1 feed (Leunisk et al.) did not affect the pH values of broiler meat [41–43].

### Effect of zeolite addition on oxidative stress

Activated and micronized zeolites are used as detoxifying agents in humans. Detoxification is attributed to their ability to reduce lipid peroxidation by scavenging free radicals [13]. In the present study, the effect of supplemental zeolite on antioxidant status of turkey are shown in Table 6a and Table 6b.

**Table 6:** Redox status in the liver (a) and the meat (b) of turkey poults fed diets with supplemental zeolite

Parameters (5a female)	BD(0%Z) Mean ± SD	BD(1%Z) Mean ± SD	BD(2%Z) Mean ± SD	*Pa-value*	*Ps-value*	
MDA (nmoles/mg prot)					0%Z VS 1%Z	0%Z VS 2%Z
female						
Female liver	1.17 ± 0.11	1.07 ± 0.19	1.00 ± 0.05	*0.171*	*0.343*	0.015*
Male liver	1.05 ± 0.11	1.00 ± 0.18	0.90 ± 0.02	*0.182*	*0.575*	0.017*
SOD (U/mg prot)						
Female liver	115.71 ± 10.89	117.82 ± 9.36	118,13 ± 10.29	*0.920*	*0.751*	*0.727*
Male liver	105.76 ± 17.71	109.88 ± 11.08	111.44 ± 10.51	*0.717*	*0.588*	*0.449*
CAT (μmole H2O2/min/mg prot)						
Female liver	0.32 ± 0.08	0.27 ± 0.13	0. 27 ± 0.03	*0.648*	*0.517*	*0.276*
Male liver	0.33 ± 0.06	0.30 ± 0.11	0. 29 ± 0.04	*0.745*	*0.663*	*0.316*
GPx (U/mg prot)						
Female liver	1.65 ± 0.25	1.81 ± 0.32	1.83 ± 0.27	*0.564*	*0.401*	*0.317*
Male liver	1.71 ± 0.26	1.75 ± 0.28	1.87 ± 0.19	*0.595*	*0.845*	*0.313*
male						
Female meat	0.62 ± 0.11	0.59 ± 0.08	0.48 ± 0.10	*0.065*	*0.621*	0.043*
Male meat	0.47 ± 0.06	0.43 ± 0.10	0.35 ± 0.06	*0.116*	*0.567*	0.023*
SOD (U/mg prot)						
Female meat	44.89 ± 2.27	46.22 ±2.07	46.49 ± 2.40	*0.506*	*0.363*	*0.311*
Male meat	43.95 ± 1.97	46.14 ± 2.86	46.71 ± 2.20	*0.196*	*0.197*	*0.071*
CAT (μmole H2O2/min/mg prot)						
Female meat	0.38 ± 0.07	0.35 ± 0.05	0.34 ± 0.04	*0.593*	*0.525*	*0.351*
Male meat	0.34 ± 0.07	0.30 ± 0.05	0.24 ± 0.04	*0.072*	*0.405*	0.043*
GPx (U/mg prot)						
Female meat	0.56 ± 0.19	0.73 ± 0.09	0.76 ± 0.18	*0.179*	*0.133*	*0.146*
Male meat	1.01 ± 0.14	1.07 ± 0.19	1.03 ± 0.09	*0.816*	*0.587*	*0.745*

*BD* basal diet, *Pa-value P-*value ANOVA, *Ps-value P-*value t-test student,

*: a significance difference between the mean/SD values,

Unit (U) represents 1 umol/L copper reducing equivalents per mg of protein in liver and meat

As they eliminate superoxide, hydrogen peroxide and hydroxyl radicals, antioxidant enzymes (SOD, GPX and catalase) play an important role in defense mechanisms against free radicals [44]. Malondialdehyde (MDA), used as the best existing measure of universal ROS, is an aldehyde produced fromlipid peroxidation, which is estimated based on its reaction with thiobarbituric acid [43]. In the present study, the addition of 2% of zeolite for experimental group significantly, (*p* < 0. 05) decrease the MDA level for female liver (1. 17 vs. 1. 00), male liver (1. 05 vs.0. 90), female meat (0.62 vs. 0.48) and male meat (0. 47 vs.0. 35) compared with the control group. In contrast, SOD and GPX are increase for meat and liver but the differences were not significant (*p* > 0.05). Moreover, dietary zeolite supplementation has an effect on CAT level (decrease from 0.341 for control group to 0.246 for experimental groups (2% zeolite)) only for male meat. The antioxidant capacity of turkey poult could be improved by supplemental zeolite. The results proved that supplementation of zeolite at all treatment concentration did not increase the activities of SOD and GPX but decrease the MDA content in meat and liver under normal condition.

There are growing evidences supporting that oxidative stress and free radical injury are the causative agents during the pathophysiological processes of alcoholic liver disease (ALD) related to excessive alcohol consumption [45, 46]. However, in our experimental model, supplemental zeolite led to a non-significant increase in liver and meat SOD activity. Some studies have shown increases SOD activity after zeolite administration [47], while others have shown decrease [48].

On the other hand, zeolite administration (5 mg/kg) orally for 10 days, in rats with partial hepatectomy decreased oxidant activity through elevation of liver tissue copper-zinc SOD activity and glutathione (GSH) levels and reduction of plasma and liver tissue MDA levels [49]. It has been reported that mice exposed to zeolite at the time of initial plaque accumulation had a significant increase in SOD activity in their hippocampi compared with age-matched control mice [50]. According to Zarcovic et al. and Basha et al. [51, 52], the supplementation of zeolite reduce lipid peroxidation levels and recovered the levels of catalase, SOD and GPX activity during pb2+ toxicity.

Recently, Hossein Nia et al. [53] have reported that zeolite (CLN) and nanoparticle of zeolite (NCLN) administration for STZ-induced diabetic rats compared with the healthy animals has no significant difference in the activity of SOD and GPX. In against part, CLN reduced MDA levels more than NCLN in the normal group (*p* < 0.05).

Zeolite was used in our study for the following reasons: zeolite is capable of adsorbing many different types of gas, moisture, petrochemicals, heavy metals and radioactive elements and a multitude of various compounds; it has a total cation-exchange capacity of about 200 mEq. The exchange capacity plays an important role in the therapeutic use of zeolite, accounting for its ability to release useful elements while capturing and binding others [54, 55].

The exact mechanisms of zeolites ‘antioxidant effects are not well known. In effect, zeolite is not absorbed into the blood from the gut. The effects of zeolite may be due to an indirect interaction with biochemical systems, removal of waste and toxins from the gut, improvement of the immune system through the mucosal-related intestinal lymphoid tissue, and an increase in the bioavailability of minerals that are important co-factors for some enzymes [56–58].

## Conclusion

The results of this study showed that the use of zeolite at a dose of 2% as feed additive had a beneficial effect on turkey’s growth, breast meat antioxidative capacity and led to the improvement of their meat quality. The present experiment shows also the significance of using zeolite, as a feed additive for turkeys, as part of a comprehensive program to increase level of polyunsaturated fatty acids on the turkey body. Hence, it is concluded that the use of alternative management strategies can include natural zeolites, however, other factors such as the type of the natural zeolite or its inclusion ratio, need further investigation.
